# UDP-glucose dehydrogenase (UGDH) in clinical oncology and cancer biology

**DOI:** 10.18632/oncotarget.28514

**Published:** 2023-09-28

**Authors:** Meghan J. Price, Annee D. Nguyen, Jovita K. Byemerwa, Jasmine Flowers, César D. Baëta, C. Rory Goodwin

**Affiliations:** ^1^Department of Internal Medicine, John Hopkins Hospital, Baltimore, MD 21287, USA; ^2^Department of Neurosurgery, Duke University Medical Center, Durham, NC 27710, USA; ^3^Department of Pharmacology and Cancer Biology, Duke University, Durham, NC 27708, USA; ^4^Department of Neurosurgery, Associated with Duke University Medical Center, Durham, NC 27710, USA; ^5^Department of Epidemiology and Clinical Research, Stanford University, Stanford, CA 94305, USA; ^6^Department of Neurosurgery, Duke Center for Brain and Spine Metastasis and Duke Cancer Institute, Durham, NC 27710, USA

**Keywords:** UDP-6 glucose dehydrogenase, UGDH, cancer, oncology, cancer biology

## Abstract

UDP-glucose-6-dehydrogenase (UGDH) is a cytosolic, hexameric enzyme that converts UDP-glucose to UDP-glucuronic acid (UDP-GlcUA), a key reaction in hormone and xenobiotic metabolism and in the production of extracellular matrix precursors. In this review, we classify UGDH as a molecular indicator of tumor progression in multiple cancer types, describe its involvement in key canonical cancer signaling pathways, and identify methods to inhibit UGDH, its substrates, and its downstream products. As such, we position UGDH as an enzyme to be exploited as a potential prognostication marker in oncology and a therapeutic target in cancer biology.

## INTRODUCTION

**Figure 1:**
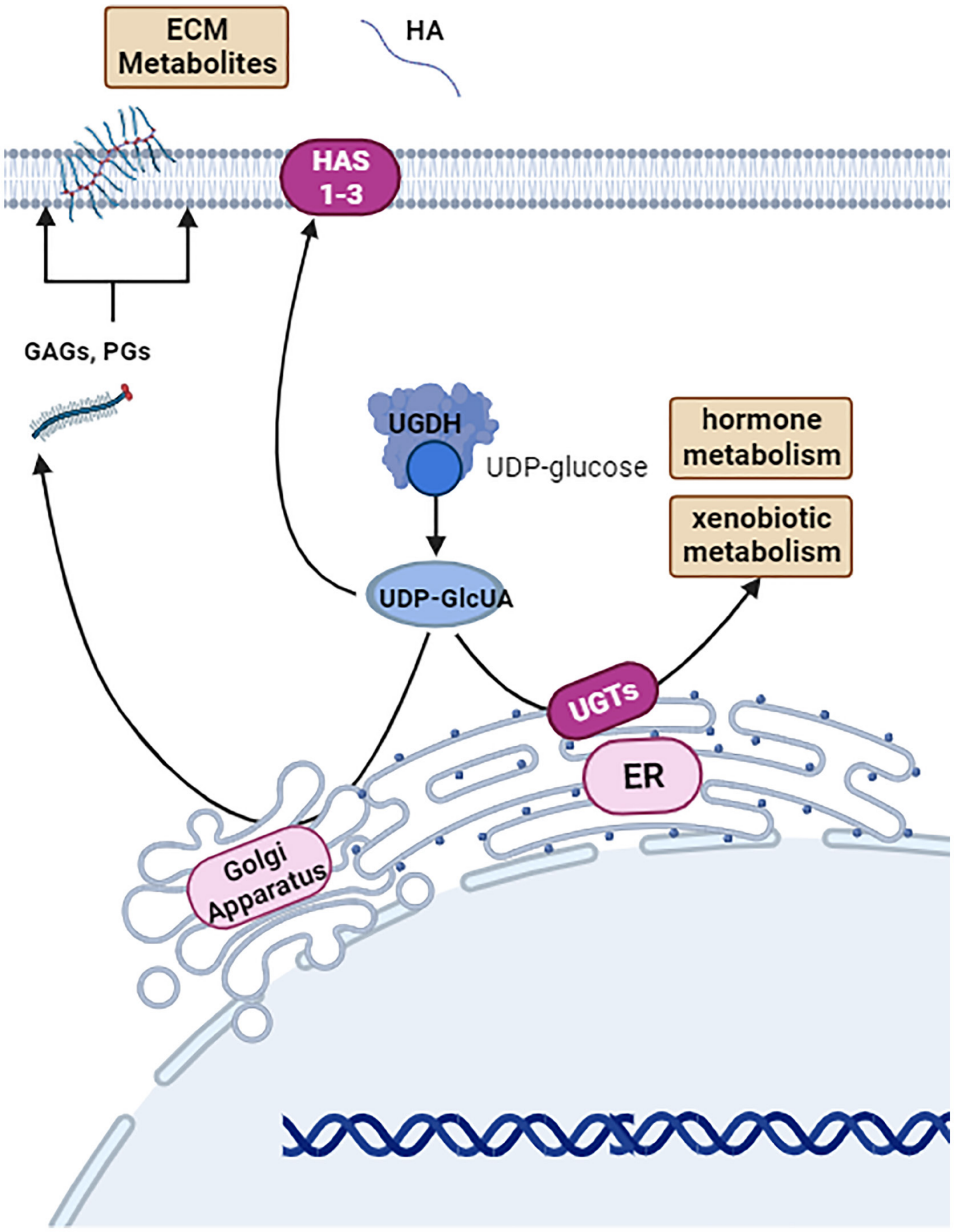
Basal function of UDP-glucose-6-dehydrogenase (UGDH). UGDH is a cystolic and nuclear hexameric enzyme that metabolizes UDP-glucose to UDP-glucuronic acid (UDP-GlcUA) as a part of xenobiotic metabolism, sugar metabolism, and generating molecule precursors for the extracellular matrix. Image was created by https://www.biorender.com (2023).

UDP-glucose 6-dehydrogenase (UGDH) is a cytosolic and nuclear hexameric enzyme that catalyzes the conversion of UDP-glucose to UDP-glucuronic acid (UDP-GlcUA). UGDH plays a role in xenobiotic metabolism via the glucuronidation pathway, sugar metabolism, production of extracellular matrix (ECM) precursors, and proteoglycan (PG) synthesis, which suggests that it may be a potential therapeutic target for a variety of diseases (Figure 1) [1, 2]. In this review, the role of UGDH in tumor progression across various cancers and the on-going efforts to pharmacologically target UGDH are discussed. Multiple studies have demonstrated UGDH’s clinical relevance to the field of oncology, and this review summarizes the evidence implicating UGDH as a candidate biomarker of aggressive cancer phenotypes and/or a potential therapeutic target to mitigate tumor progression and enhance patient survival.

### The role of UGDH in oncology

The importance of UGDH in human cancer is a topic of recent interest, both from a mechanistic and prognostication standpoint [3–6]. This section will delineate what is known about the role of UDGH across different human cancers including lung, breast, esophageal, hepatocellular carcinoma (HCC), prostate, ovarian, colorectal and melanoma with a focus on mechanistic categories (summarized in Table 1).

**Table 1:** Studies that discuss the role of UGDH in oncology categorized by tumor type

Study	Cancer type	Cell line studied	Mechanisms discussed	Primary findings
Paul et al., 2016	Lung (NSCLC)	• NCI-H460 • NCO-H460R	• Chemotherapy (etoposide) resistance	• Etoposide treatment led to alteration of 83 proteins in NSCLC lung cancer cell lines (UGDH included) • UGDH not functionally a/w chemotherapeutic resistance
Wang et al., 2019	Lung (adeno)	• A549 • H1299 • PC-9	• EMT factor stabilization • Downstream signaling pathways	• Higher levels of activated (phosphorylated) UGDH associated with increased stability of EMT transcription factor SNAI1 • UGDH activation induced by EGF binding to EGFR • Inhibiting UGDH *in vitro* and *in vivo* decreased migratory and metastatic phenotypes; higher levels of pUGDH in patient associated with higher mortality • MEK inhibitor abrogated EGF induced pUGDH
Hagiuda et al., 2019	Lung (adeno)	• LC-2 • A549	• Cellular localization	• Nuclear localization of UGDH associated with more aggressive, migratory phenotypes • Worse survival in patient samples with nuclear localization of UGDH
Richter et al., 2021	Lung (SCC*) Head and neck (SCC)	• NCI-H2170 (lung) • PCI-13.1 (H/n)	• Differential expression across cancer type	• UGDH upregulated in squamous cell cancer lung cancer • Levels of UGDH not different enough between SCC lung and head/neck cancer to distinguish them
Arnold et al., 2019	Breast (TNBC)	• MDA-MB-231	• EMC modulation • HA regulation • Lipid metabolism	• Higher levels of UGDH in more invasive malignant BC patient samples • Overexpression of EMT TF increased expression of UGDH • Depleting UDP-GlcUA inhibited mesenchymal phenotypes including cellular invasion and colony formation *in vitro* and metastatic phenotype *in vivo* • HA rescued UGDH KD phenotype • Fatty acid metabolism and PPAR-gamma pathway altered by UGDH KD
Teoh et al., 2021	Breast (TNBC)	• 6DT1	• UDP glucose metabolism • Downstream signaling pathways	• High UGDH expression associated with worse patient survival • UGDH KD decreased migratory and metastatic phenotype of BC cells *in vitro* and *in vivo* • High UGDH a/w TP53 mutations and copy number alterations in BRCA1 and PIK3CA
Vitale et al., 2021	Breast (TNBC)	• MDA-MB-231	• Chemotherapy (epirubicin resistance) • HA • ECM modulation	• Higher levels of UGDH correlated with worse prognosis in patients with TNBC who received chemo • UGDH KD associated with epirubicin resistance • UGDH KD resulted in increased epirubicin accumulation, increased apoptosis and positive modulation of autophagy • Epirubicin resistance potentially related to HA metabolism and ECM modulation
Lin et al., 2020	Ovarian	• TOV21G	• EMT • Downstream signaling	• UGDH overexpressed in ovarian cancer tissue • UGDH KD decreased metastatic ability *in vitro* and *in vivo* • UGDH depletion down-regulated EMT markers and ERK/MAPK pathway
Liu et al., 2020	Esophageal	• Human samples	• UGDH expression	• UGDH-AS1 (lncRNA) levels correlated with overall survival in patients • UGDH-AS1 had low expression level in samples • UGDH mRNA not a/w patient survival
Oyinlade et al., 2018	Brain (GBM)	• U87	• ECM modulation	• KLF4 upregulates UGDH expression by methylating CpGs • UGDH required for KLF4-induced cell migration • UGDH KD decreases GAG abundance, cell proliferation and migration *in vitro* and tumor growth/ migration *in vivo* • UGDH KD a/w decreased expression of ECM proteins tenascin C, brevican
Wei et al., 2009	Prostate	• LNCaP C33 (low passage) • LNCaP C81 (high passage)	• Hormone metabolism • ECM modulation	• Dihydrotestosterone (DHT) increases UGDH expression in androgen-dependent cells • Increased metabolism of DHT in AD cells than non-AD cells • Increased DHT metabolism corresponded to slower cellular growth
Huang et al., 2010	Prostate	• Human samples	• UGDH expression	• Higher UGDH expression in cancerous acini compared to noncancerous controls
Zimmer et al., 2016	Prostate	• LNCaP (AD) • LNCaP 81 (CR)	• Hormone metabolism • ECM modulation	• CR tumor cells express higher levels of UGDH • AR-dependent expression of PSA and UGDH downregulated in CR cells • UDP-sugar flux increase through PG and GAG synthesis pathways rather than glucuronidation
Zimmer et al., 2021	Prostate	• LNCaP (AD) • LNCaP (CR)	• Hormone metabolism • ECM modulation	• Overexpression of UGDH in AD cells blunted androgen-dependent gene expression, increased PG synthesis, and increased migratory phenotype • Overexpression of UGDH decreased growth suppression seen with enzalutamide • UGDH KD decreased PG production, restored AD, and sensitized cells to enzalutamide
Wang et al., 2010	Colorectal Cancer (CRC)	• HCT-8	• ECM modulation	• UGDH KD associated with decreased UDP-GlcUA and GAG production • Treatment with 4-MU decreased cell aggregation and motility *in vitro* • Cell aggregation and migration restored with exogenous HA
Shen et al., 2016	Colorectal cancer (CRC)	• Human samples	• ECM modulation • Cell metabolism	• Assessed differentially expressed genes (DEGs) in CRC samples • UGDH identified in a network of genes functionally associated with metabolism-related functions
Deen et al., 2016	Melanoma	• MV3 • C8161	• ECM modulation • Glucose metabolism	• Recycling of HA synthesis enzymes controlled by cytosolic levels of UDP-GlcUA and UDP-GlcNAc • Lower levels of UDP-GlcUA and UDP-GlcNAc inhibits HA synthesis • Correlation between HA content in human melanoma samples
Fan et al., 2009	Hepatocellular Carcinoma (HCC)	• LCI-D20	• Drug mechanism (cell differentiation agent-II CDA-II)	• CDA-II suppresses growth and metastasis • 27 genes including UGDH differentially expressed in response to treatment with CDA-II • UGDH downregulated in response to CDA-II • Downstream genes c-myc, N-ras, and MMP-9 down-regulated

### UGDH as a prognostication marker of tumor progression

UGDH became an oncologic target of interest in the early 2000s primarily within breast (BC) and prostate cancer (PC) research [7, 8]. Several additional studies in BC have further established that higher levels of UGDH are associated with worse prognoses for patients (particularly those with triple negative breast cancer (TNBC) receiving chemotherapy [5]) and more invasive, metastatic phenotypes in BC samples [9, 10]. Similar findings are demonstrated in lung cancer; specifically in 2019, Wang et al., defined the role of UGDH in promoting the stability of epithelial-to-mesenchymal transition (EMT) factors in lung adenocarcinoma. They also reported that patients with tumors expressing higher levels of phosphorylated UGDH (specifically Y473) had lower median survival than those who did not [11]. While UGDH phosphorylation has not been mechanistically explored in other cancers or reported in normal physiology, this study did correlate phosphorylated UGDH to a metastatic and pro-EMT phenotype of lung adenocarcinoma. In contrast, higher levels of UGDH are not ubiquitously associated with worse prognoses in all human cancer patient samples; for example, within esophageal cancer, there are conflicting findings. Liu et al., 2020 explored the prognostic value of lncRNA and found that UGDH-AS1 had lower expression levels in esophageal cancer samples while Luo et al., 2021 did not find a correlation between mRNA levels of UGDH and prognosis/survival for patients with esophageal cancer [12, 13]. For prostate cancer, higher levels of UGDH have been observed in cancerous prostate acini than non-cancerous prostate tissue—a finding that established UGDH as a potential biomarker for PC [8].

### UGDH regulation of the extracellular matrix (ECM) and hyaluronic acid production

UGDH catalyzes conversion of UDP-glucose to UDP-GlcUA to generate proteoglycans (PG) and glycosaminoglycans (GAGs) for the ECM. PGs and GAGs are building blocks of the extracellular matrix (ECM) and are critical metabolites in normal cellular structure and function such as wound healing, immune system processes, chondrogenesis, and embryonic development [2, 14, 15]. One GAG molecule of particular importance is hyaluronan or hyaluronic acid (HA) due to its abundance in the ECM and role in cell differentiation, survival, angiogenesis, and tumor formation [16, 17]. The importance of the ECM in tumor progression, growth, and migration has become increasingly complex as our understanding of the tumor microenvironment expands. The role of UGDH and its downstream product UDP-GlcUA in producing ECM precursors HA, proteoglycans (PGs) and other glycosaminoglycans (GAGs) is particularly salient to studies assessing tumor aggression and migratory capacity. These ECM precursors, specifically HA, have been implicated in worse patient prognoses, metastatic phenotypes, and higher proliferation/migratory capabilities of cancer cell lines *in vitro*. Independent of UGDH, high HA levels are considered a marker of malignancy in several types of solid tumors including melanoma, bladder, lung, prostate, breast, and colon [18–23]. HA is shown to regulate the tumor microenvironment by promoting cell adhesion, migration, and proliferation via signal transduction and interaction with different receptor signaling pathways. In doing so, HA promotes an invasive/metastatic phenotype through induction of EMT promoting pathways [24, 25]. As a necessary element in HA formation, UGDH knock down (UGDH KD) is shown to decrease HA formation and thus alter the associated aggressive tumor microenvironment phenotype [4, 9].

Within the field of breast cancer research, the role of HA in relation to UGDH has been primarily studied in TNBC. Arnold et al., 2019 demonstrated that depleting UDP-GlcUA (via knocking down UGDH) was sufficient to decrease HA production and thereby inhibit invasion, colony formation, and tumor growth both *in vitro* and *in vivo*. When HA was added back to these experiments, they were able to rescue 80 to 90% of the migratory phenotype, suggesting that these findings were significantly dependent upon HA production.

In a different study focused on the potential role of UGDH expression and HA metabolism in epirubicin resistance in TNBC, the authors found that while UGDH expression was correlated with worse prognosis, UGDH KD contributed to drug resistance. This was surprising as knocking down UGDH inhibited glucuronidation, which is responsible for the cellular elimination of epirubicin; and thereby increased intra-cellular epirubicin levels. Paradoxically, this did not increase cytotoxicity; rather, UGDH KD was associated with increased autophagy of cancer cells, which is involved in the development of epirubicin resistance [5]. The authors also observed that UGDH KD in combination with epirubicin treatment was associated with modulation of HA, HA synthesis (HAS) enzymes, and HA degrading enzymes synthases (HYAL). These enzymes are responsible for HA turnover; the authors found that more deposition and catabolism of HA resulted in a more resistant phenotype [5]. From this observation, they proposed that UGDH KD could produce an ECM with abnormal HA production and metabolism that ultimately favors treatment resistance. Thus, while there are demonstrated relationships between UGDH and HA in breast cancer in experimental settings, these relationships are less well studied in clinical practice.

The relationship of UGDH and HA metabolism has also been shown to play a role in tumor aggressiveness in melanoma, colorectal cancer, nasopharyngeal and primary brain tumors. In their 2016 study, Deen et al., established the importance of UD-GlcUA and UDP-GlcNAc levels to the intracellular movement and processing of hyaluronan synthases 1–3 (HAS1-3) for melanoma. Specifically, lower levels of UDP-GlcUA resulted in more HAS endocytosis and therefore inhibition of HA synthesis via a regulatory feedback cycle. UGDH was critical to this cycle as the authors demonstrated that decreasing its expression and activity correlated with lower levels of both UDP-GlcUA, and HA. Within tissue samples, the authors also correlated levels of hyaluronan and UGDH mRNA with different stages of melanoma development and thus suggested that UDP-sugar metabolism is critically linked with hyaluronan and may support progression of melanoma [26]. Similar phenotypic findings have been reported in colorectal cancer with UGDH KD effectively decreasing cell migration and motility in both transwell migration assays and 3-D collagen gels [27]. The authors were able to rescue the migratory phenotype with subsequent treatment of the colorectal cells with HA *in vitro* [27]. Within nasopharyngeal carcinoma, (a tumor with high metastatic potential due, in part, to high expression levels of Epstein-Barr virus latent membrane protein 2A (LMP2A)), LMP2A-induced higher expression of UGDH, subsequently increased GAG synthesis [28]. They were able to modulate this activation pathway by overexpressing or inhibiting specificity protein 1 (Sp1) upstream of LMP2A and UGDH [28].

The relationship of elevated GAG formation to more aggressive phenotypes in primary brain tumors (specifically glioblastoma multiforme (GBM)) had been well established when Oyinlade et al., 2018 directly linked upregulation of UGDH expression to increased GAG levels in GBM. Methylation of Kruppel-like factor 4 (KLF4) upregulated the expression of UGDH resulting in higher intra-tumoral levels of GAGs and thereby increased proliferation and migration of GBM cell lines. Subsequently, knocking down UGDH *in vitro* and *in vivo* abrogated this aggressive phenotype while decreasing the expression of specific ECM proteins (tenascin C, brevican) [4]. Thus, multiple cancer models demonstrate a direct link between UGDH activity, ECM precursor formation, and subsequent aggressive and metastatic oncologic phenotypes.

### UGDH regulation of EMT in metastasis

Closely linked to HA production and ECM modulation is the role of UGDH in regulating genes responsible for the epithelial to mesenchymal transition (EMT). This process has been well studied in lung, breast, ovarian, brain, and colon cancer. In a landmark paper on the role of UGDH in lung cancer, Wang et al., 2019 directly linked UGDH with enhanced mRNA stability of the EMT factor SNAI1. This connection mechanistically explained the increased *in vitro* and *in vivo* tumor cell migration and metastasis associated with higher levels of UGDH in lung adenocarcinoma. Of note, this interaction was dependent on EGFR-mediated attachment of the RNA binding protein HuR to phosphorylated UGDH. Subsequently this phosphorylated UGDH was responsible for converting UDP-Glc (which prevents HuR from binding to mRNA) to UDP-GlcUA, allowing HuR to bind to and stabilize SNAI1 mRNA and promoting an aggressive EMT phenotype [11]. While not as clear mechanistically, UGDH KD has also been shown to decrease expression of EMT transcription factors SNAIL, SIP-1, and matrix mellatoprotease protein 2 (MMP2) in ovarian cancer cell models. In these ovarian cancer models, knocking down UGDH also decreased the activity of actin as a key migratory protein. While the mechanism for increased mRNA stability presented by Wang et al., 2019 provides potential mechanism for UGDH’s role in pro-EMT phenotypes, there are likely many other mechanisms contributing to this process across various cancers.

UGDH-mediated EMT gene expression is not shown to consistently modulate migratory phenotypes across all tumor types. For example, in breast cancer, Teoh et al., 2020 demonstrated that increased UGDH levels were significantly associated with more aggressive migratory phenotypes; however, many EMT genes were not transcriptionally inhibited by decreased UGDH expression [10]. Paradoxically, both fibronectin (Fn1) and Six1, glycoproteins in the extracellular matrix associated with more aggressive breast cancer phenotypes, were upregulated in UGDH KD. This suggests that EMT gene modulation was not responsible for the observed more aggressive tumorigenic phenotypes in this study. One study also suggested that rather than activating EMT, UGDH activation and subsequent ECM remodeling may be downstream of EMT initiation as the authors demonstrated that overexpressing EMT transcription factors SNAI1 and TW1ST induced UGDH expression [9]. This increased UGDH expression in turn increased flux through HA and UDP-sugar pathways [9]. They proposed that rather than it being the promoter of EMT, UGDH was a critical enzyme in the glucose metabolic reprogramming that accompanies the EMT process [9].

### The role of UGDH in cancer biology

#### UGDH’s role in canonical cancer signaling pathways

**Figure 2:**
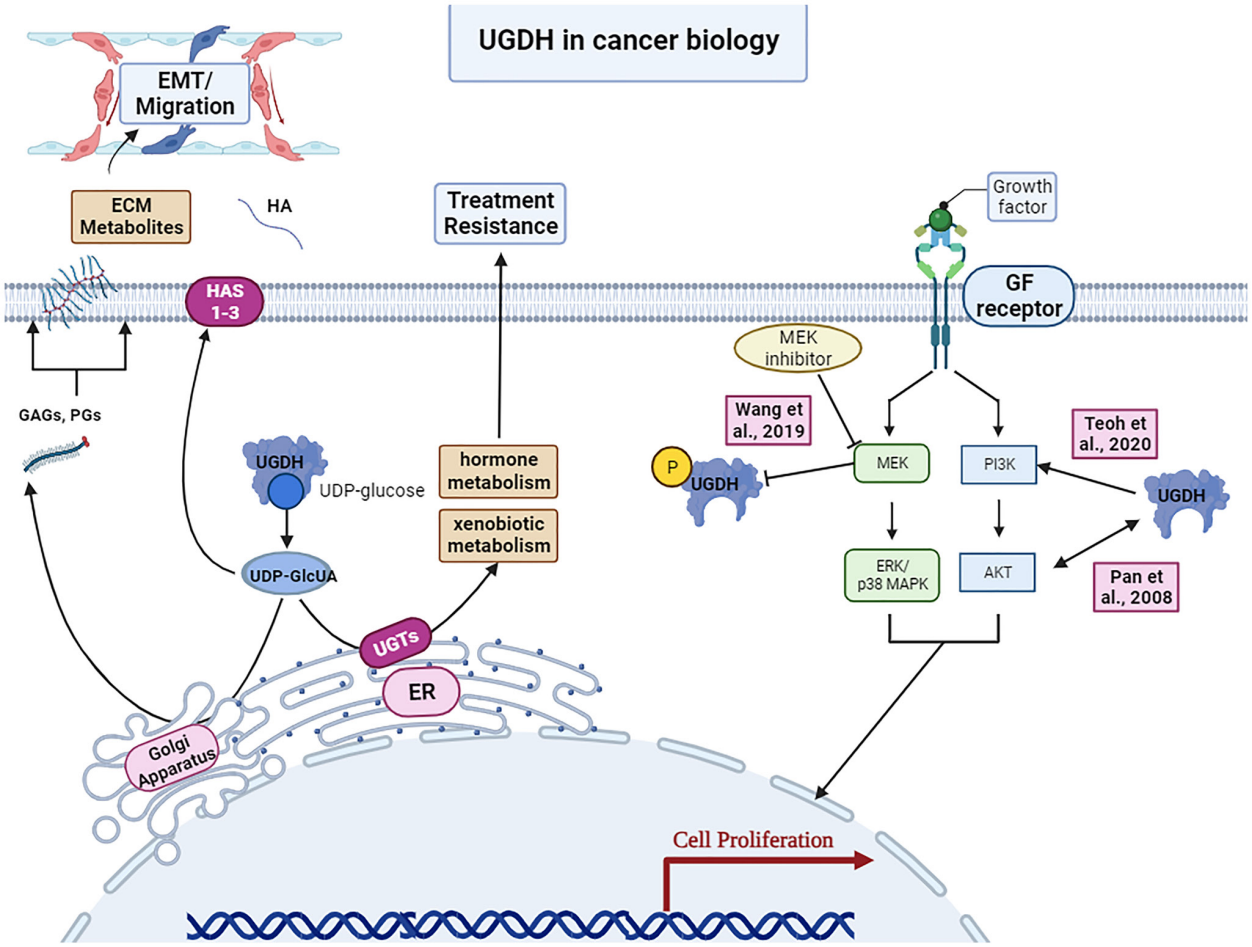
UGDH’s roles in cancer biology. Upregulation of UGDH’s generation of ECM metabolites can contribute to epithelial mesenchymal transition and migration in metastasis. Hormone metabolism and xenobiotic metabolism by UGDH may promote treatment resistance. UGDH has also been shown to interact with the MEK/ERK and PI3K/AKT pathways of canonical growth factor signaling pathways in cancer biology. Image was created by https://www.biorender.com/ (2023).

To better identify mechanistic similarities of UGDH amongst various cancers, it is critical to understand the effect of modulating UGDH on downstream signaling pathways. While many of these relationships are not fully understood, several studies outlined previously have described connections between intracellular signaling enzymes such as MAPK, ERK, and AKT that provide a groundwork for our understanding of how UGDH affects these intracellular processes (Figure 2) [10, 28]. Furthermore, it has been well established that MAPKs are associated with cancer cell proliferation, survival, metastatic capacity, and motility [29].

Within lung cancer, Wang et al., 2019 explored the effects of inhibiting downstream targets within the EGFR induced MAPK pathway on the tumorigenic phenotype induced by phosphorylated UGDH (pUGDH). They found that the MEK inhibition but not p38 MAPK inhibition was able to abrogate EGF-induced pUGDH; additionally, knocking down UGDH did not affect ERK phosphorylation. Interestingly, in ovarian cancer, UGDH KD has been shown to negatively regulate levels of activated (phosphorylated) ERK with direct impacts on expression of MMP-2, MMP-9 and EMT-related factors further downstream [3]. These findings parallel those of Clarkin et al., 2011: while normal UGDH expression and function appear to be p38MAPK pathway dependent, stimuli activating UGDH (such as growth factors, induced overexpression, and phosphorylation as in Wang et al., 2019) shifts intracellular signaling towards a more MEK/ERK dependent mechanism [11, 14]. The AKT pathway has also been a target of interest within UGDH research. In breast cancer, Teoh et al., 2020 commented on an observed association between lower UGDH expression and copy number alterations in PIK3CA which produces PI3K, a component of the AKT signaling pathway. While they could not mechanistically explain this observation, it is nevertheless interesting as the AKT pathway has been shown to regulate UGDH expression in colorectal and nasopharyngeal cancer [28, 30]. Specifically, in nasopharyngeal cancer, Pan et al., 2008 demonstrated that UGDH expression induced PI3K/AKT and ERK activity [28] while Haggblad showed that AKT KD resulted in decreased expression of UGDH [30]. Thus, results of these studies both indicate the importance of these downstream signaling pathways and suggest that the relationship between them and UGDH is likely complex and bi-directional.

#### UGDH’s role in hormone metabolism in hormonally responsive cancers

Along with its relationship to downstream signaling pathways, UGDH has a key role in glucose/UDP-sugar metabolic pathways associated with cancer progression. As mentioned previously, both UGDH and HA levels have been connected to reprogramming glucose metabolism in breast cancer. Further, Arnold et al., 2019 demonstrated that changes in glucose metabolism via UGDH KD were also associated with differential essential of genes involved in fatty acid/lipid metabolism and PPAR signaling [9]. Within colon cancer, Shen et al., 2017 performed a study to identify differentially expressed genes (DEGs) in patient samples compared to non-cancerous controls and found that UGDH was key to a network of genes that regulated cancer cell metabolism (UGDH, ALDH1A1, FABP4, and MGLL). The specific mechanism was not identified; however, it was clear that expression of these genes along with several others involved in ECM regulation (COL1A1, COL1A2, and MMP9) were critical to the tumorigenesis of the CRC samples in this study [31]. Similarly, within melanoma, the synthesis and turnover of HA was directly linked to the downstream products of UDP-sugar metabolism—a relationship that together may be responsible for initiating and supporting the progression of melanoma [26].

In addition to the effect of UGDH on metabolic pathways, its previously discussed role in glucuronidation is important for regulating levels of intracellular hormones. Interestingly, the relationship between UGDH and hormones may be bi-directional as UGDH expression can be increased with exposure to certain endogenous hormones such as estradiol and dihydrotestosterone (DHT) [32]. Most studies exploring the relationship amongst UGDH, glucuronidation, and cancer have been conducted in hormonally responsive cancers such as prostate and breast. Specifically for prostate cancer, the leading hypothesis is that differential expression of UGDH can modulate how UDP-sugars flux through hormonal processing and ECM production pathways [32]. UGDH regulation of glucuronidation therefore could be a mechanism to promote androgen response deregulation and increase castration resistance within hormonally responsive prostate cancer. Mechanistically, Zimmer et al., 2016; 2021 have proposed that knocking down UGDH inhibits glucuronidation within cells and thereby decreases excretion of androgens, allowing for higher intracellular levels of this tumorigenic hormone. This is, in turn, accompanied by an increased flux of UDP-sugars through alternate UGDH mediated pathways within the cells to produce proteoglycans responsible for increased migratory phenotypes [32]. Indeed, the authors demonstrated that over-expressing UGDH in both androgen responsive and castrate resistant prostate cancer cell lines can induce androgen independent growth. Furthermore, loss of UGDH promoted androgen receptor dependent gene expression and restored androgen sensitivity to castrate resistant cells. This shift was accompanied by a decreased production of PGs [2]. These findings suggest that paradoxically, within certain hormonally responsive tumors, loss of UGDH may be associated with a more aggressive phenotype given its role in endogenous hormone processing. These findings highlight the importance of understanding the role of UGDH generally within in cancer but also emphasize how critical it is to study the nuances of UGDH function within specific cancers and environments.

### UGDH as a therapeutic target

Given the well-established association between high levels of UGDH and aggressive, metastatic tumor phenotypes across many cancers, there is significant potential to use UGDH as both a prognostic marker and therapeutic target in clinical settings. As a catalyst of rate-limiting steps in several pro-tumorigenic pathways within cancer cells, UGDH, along with its substrates and downstream products, present promising targets for drug discovery.

There are multiple potential strategies to target either UGDH and/or up- or downstream pathways. A direct inhibitor of UGDH would be the most effective way to target its enzymatic activity; however, given the ubiquitous presence of UGDH in cells, specifically targeting the pro-tumorigenic effects of UGDH could be challenging. Indeed, there are no specific small molecule inhibitors of UGDH currently available in clinical settings. Alternatively, the UGDH pathway could be targeted by depleting substrates such as UDP-glucose or inhibiting its downstream products such as UDP-GlcUA or GAGs/HAs. In this section, we will discuss past and ongoing efforts to develop UGDH small molecule inhibitors along with therapeutic strategies to target substrates and downstream products (summarized in Table 2).

**Table 2:** Studies that discuss therapeutics targeting UGDH categorized by mechanism of action

Therapeutic molecule	Associated study/studies	Classification	Mechanism of action
**UDP-7-deoxy-α-D-gluco-hept-** **6-ulopyranose (ketone 5)**	Campbell and Tanner, 1999	Direct competitive inhibitor of UGDH	Carbonyl functionality at C6 forms a carbon-carbon bond with UGDH and prevents second oxidation reaction (K_*i*_ value of 6.7 uM)
**UDP-a-D-xylose (UDX)**	Neufeld 1965 Kadirvelraj et al., 2014 Sennett and Wood, 2012	Allosteric inhibitor of UGDH	UDX binds to the active site of UGDH to increase the affinity between UGDH subunits and occlude binding sites for cofactor NAD+ to inactivate UGDH
**Polyphenols (gallic acid,** **Quercetin)**	Hwang et al., 2008	Indirect target of UGDH (reduces activity of UGDH)	Inhibition of proliferation (exact mechanisms unknown)
**Aphanizomenon flos-aquae ** **(AFA)**	Scoglio et al., 2016	Antioxidant acting as a mixed-type inhibitor	Inhibits binding of both UDP-Glc and NAD+
**5-hexyl-2-deoxyuridine ** **(HUdR)**	Lapis et al., 1987 Jeney et al., 1990 Timar et al., 1990 Pogany et al., 1990 Harisi et al., 2009	Inhibition of UGDH substrate synthesis	Inhibits conversion of glucosamine to UDP-sugars to deplete UDP-glucose
**4-Methylumbelliferone (4-MU) ** **OR Hymecromone**	Yoshihara et al., 2005 Nakazawa et al., 2006 Lokeshwar et al., 2010 Arai et al., 2011 Twarock et al., 2011 Urakawa et al., 2012 Zhan et al., 2022	Inhibition of UGDH downstream metabolites	Glucuronidation of 4-MU depletes cellular UDP-GlcUA to prevent HA synthesis

#### Targetable structural properties of UGDH

**Figure 3:**
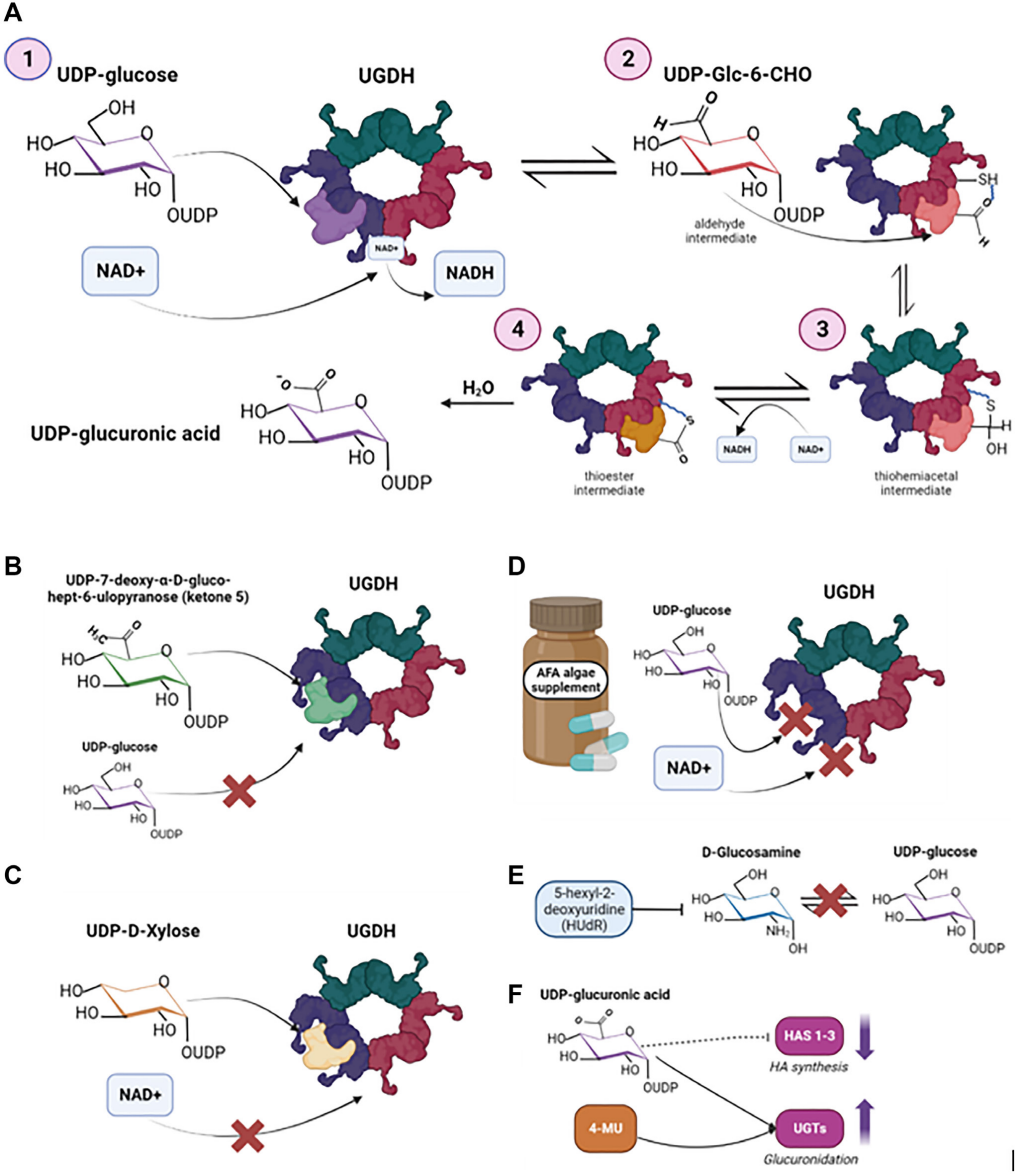
UGDH biochemical mechanism and therapeutic targets that exploit the structure of UGDH. (**A**) Metabolism of UDP-6-glucose to UDP-GlcUA by UGDH proposed by Ridley et al., 1975. (**B**) UDP-7-deoxy-α-D-gluco-hept-6-ulopyranose (ketone 5) as a direct competitive inhibitor of UGDH, as proposed by Campbell and Tanner, 1999. (**C**) UDP-D-xylose (UDX) as an allosteric inhibitor of UGDH, preventing the binding and reduction of NAD+. Neufeld, 1965, introduced UDX as an endogenous feedback inhibitor of UGDH. (**D**) Aphanizomenon flos-aquae (AFA), an algae supplement that acts as a mixed-type inhibitor and prevents the binding of both UDP-glucose and NAD+, as discussed by Scoglio et al., 2016. (**E**) 5-hexyl-2-deoxyuridine (HUdR) as an inhibitor of UGDH’s substrate UDP-glucose, by preventing the conversion of glucosamine to UDP-sugars to ultimately prevent the biosynthesis of heparan sulfate. (**F**) 4-Methylumbelliferone (4-MU) or Hymecrome as a HA synthesis inhibitor, by depleting UDP-GlcUA. Image was created by https://www.biorender.com (2023).

Structurally, UGDH is most stable as a hexameric quaternary structure, as identified through X-ray crystallography [33–35]. Its most common form is a 57kDa hexamer assembled into a trimer of dimers, in which only three subunits are simultaneously active [1, 36, 37]. Enzymatic activity is controlled by induced fit responses that regulate subunit affinity and quaternary structure, and there is a single allosteric and active site [38]. While there is consensus that UGDH catalyzes UDP-glucose, the mechanism by which this catalysis occurs remains controversial and ambiguous despite extensive investigations. In the classic mechanism proposed by Ridley et al., the catalysis follows 4 steps. First, UDP-glucose is converted to an aldehyde intermediate, the UDP-*α*-D-*gluco*-hexodialdose (UDP-Glc-6-CHO) and NADH+ through a transfer of pro-R hydride to NAD+. The second step involves an attack of UDP-Glc-6-CHO by a cysteine residue in UGDH active site, leading to the formation of a thiohemiacetal intermediate that is covalently bound to the enzyme (Figure 3A). This reaction takes places immediately after the first oxidation, and it is the reason why the aldehyde intermediate isn’t released. In the third step, the thiohemiacetal is then oxidized to a thioester intermediate, resulting in the formation of a second NADH molecule. Finally, an irreversible hydrolysis of the thioester takes place to yield UDP-GlcUA [39]. Skeptics of this model argue that experimental evidence do not suggest the presence of a bound intermediate. Alternatively, it has been argued that UGDH converts to UDP-glucose to a Schiff base instead of an aldehyde intermediate [40], while more recent literature suggests a model in which the first oxidation step bypasses the aldehyde via an NAD+ dependent biomolecular nucleophilic substitution [41]. Despite these controversies regarding the first steps of this catalytic process, there is a consensus on the enzymatic steps after the first oxidation. The UGDH enzyme has a high affinity for allosteric inhibition. Due to its intrinsically disordered carboxy terminus (ID-tail), UGDH is highly regulated by allosteric binding of its downstream products including UDP-xylose (UDX), UDP-GluCA and other co-enzymes (NAD+), via feedback inhibition [36]. In most cases, UGDH is regulated by an atypical allosteric mechanism, in which UDP-Xyl competes with UDP-glucose for the active site, converting UGDH into an inactive state or inducing hysteresis or a “lagging” metabolism, which could have potential therapeutic implications [42].

#### Direct targets of UGDH

##### UDP-Glucose Analogues as inhibitors of UDGH

Certain aspects of UGDH’s enzymatic activity present potential targets for drug discovery. Specifically, targeting the bound aldehyde intermediate produced in the first step of UGDH’s enzymatic reaction has been previously explored [43]. Leveraging this step in the enzymatic activity of UGDH, Campbell and Tanner, 1999 designed UDP-glucose analogues that mimicked the bound aldehyde intermediate and thereby bound more tightly to UGDH than its natural substrate UDP-glucose. Using this strategy, they synthesized and tested UDP-7-deoxy-α-D-gluco-hept-6-ulopyranose (ketone 5) whose carbonyl functionality at C6 led to a formation of a carbon-carbon bond with UGDH, which prevented the second oxidation reaction from proceeding (Figure 3B). They found that this compound could act as a competitive inhibitor of UGDH with a K_*i*_ value of 6.7 uM [43]. While this analogue was promising, there have not been follow-up studies to test its *in vitro* and *in vivo* efficacy or phenotypic impact.

##### UDP-a-D-xylose (UDX)

Apart from its roles in drug metabolism and ECM maintenance, UDP-GlcUA is a precursor for the biosynthesis of UDX, an endogenous feedback inhibitor of UGDH (Neufeld 1965). UDX differs from UDP-GlcUA as it lacks a C’ hydroxymethyl group. Thus, when UDX binds the UGDH enzyme in the active site, it triggers an allosteric switch that increases the affinity between the subunit interfaces, resulting in a hexamer that occludes the binding sites for the cofactor (NAD+) and the C5’hydroxymethyl on the substrate to inactivate the enzyme [44]. The crystal structure of UGDH bound to UDX was published in 2011 [45]. While UDX has not been formally studied as a therapeutic inhibitor of UGDH, the structural and enzymatic impacts of its binding to UGDH could be utilized to design mimetics of UDX for clinical use (Figure 3C).

#### Indirect therapeutic targets of UGDH

While there are not many, some studies have presented alternative strategies to inhibit UGDH activity in cancer cells. Specifically, Hwang et al., 2008 demonstrated the inhibitory effects of two polyphenols (Gallic acid and Quercetin) against UGDH in MCF-7 breast cancer cells [46]. They found that treating the cells with 300 uM of gallic acid reduced the specific activity of UGDH by 66% in comparison to control while treating with quercetin reduced the specific activity by 41%. While they were able to show that these compounds inhibited proliferation of MCF-7 cells, the study was limited as they could not directly link inhibition of UGDH to the anti-proliferation activities of these polyphenols. Additionally, the doses used in the study were 10-fold higher than what could realistically be achieved through diet. Despite these concerns, the structure of these polyphenols could be used for rational design of better compounds that could bind and inhibit UGDH is an effective manner and at lower doses.

Another study assessed the efficacy of the dietary supplement Aphanizomenon flos-aquae (AFA), which has strong antioxidant activity. They found out that AFA reduced UGDH activity in a dose-dependent manner and acted as a mixed-type inhibitor with respect to both UDP-Glc and NAD+ (Figure 3D). Phenotypically, they showed that AFA was also effective in reducing the colony formation capacity of PC-3 prostate cancer cells and FTC-133 thyroid cancer cells [47].

#### Inhibiting the UGDH pathway

##### Substrate inhibition upstream of UGDH

Along with directly inhibiting the enzymatic activity of UGDH, depleting its UDP-glucose substrate could be an effective strategy to target the UGDH pathway. 5-hexyl-2-deoxyuridine (HUdR) inhibits the conversion of glucosamine to UDP-sugars, which depletes UDP-glucose and subsequently reduces the biosynthesis of heparan sulfate (Figure 3E) [48, 49]. In the context of tumor biology, HUdR has been shown to reduce the migratory capacity of various tumor cells [50, 51] and has been tested *in vivo* as an anti-metastatic drug with efficacy in tumor cells with high metastatic potential [48, 52]. While these studies indirectly discuss UGDH as a therapeutic target, their findings demonstrate the potential anti-tumorigenic effects of targeting the availability of one of its substrates. Further work is necessary to follow up on the use of HUdR or similar therapies in the clinic.

##### Inhibition of UGDH products

Strategies that deplete UGDH’s downstream metabolite, UDP-GlcUA could mitigate the pro-metastatic, migratory phenotype induced by higher levels of UGDH. One of the most used drugs for this purpose is 4-MU, which results in depleted HA production downstream of UGDH. Mechanistically, glucuronidation of 4-MU depletes cellular UDP-GlcUA stores necessary for HA synthesis (Figure 3F) [18, 53–55]. 4-MU is non-toxic and non-polar which allows it to cross the lipidic intestinal barrier and thereby be administered orally. Studies have established the safety of 4-MU in humans when used as a choleretic and spasmolytic to increase bile production and thereby improve liver detoxification. Extensive *in vitro* and *in vivo* studies have shown that 4-MU reduces the proliferation, migration, invasion, and metastasis of multiple cancer cell types including pancreatic, prostate, melanoma, esophageal, breast, liver, bone and ovarian cancers [18, 53, 54, 56, 57]. Additionally, 4-MU inhibition of HA has been shown to increase access to drugs and immune infiltration to the tumor, which in turn prevent tumor growth and metastasis [55]. This was demonstrated by Nakazawa et al., 2006 who showed that, while 4-MU by itself did not cause cancer cell death or inhibition of proliferation of a pancreatic cancer cell line KP1-NL, 4-MU pretreatment increased the efficacy of an anti-cancer agent gemcitabine [55]. Additionally, 4-MU and UGDH knockdowns were shown to increase anti-tumor immune responses in GBM *in vitro* and *in vivo* by activating phagocytosis in tumor-residing macrophages, decreasing immune-suppressing regulatory T-cell activity, and increasing cytotoxic T-cell infiltration and activation [6]. This could mean that targeting the UGDH-HA pathway might expose therapeutic vulnerabilities of various cancers and lead to more efficient combination therapies involving both chemotherapies and immunotherapies.

In addition to the mechanism discussed above, recent literature has demonstrated potential direct effects of 4-MU on UGDH itself. Using limb bud micro mass cultures, Clarkin et al., 2011 found that 4-MU treatment reduced UGDH mRNA and HAS-2 expression in AS cells and produced modest suppression of UGDH protein levels. This was subsequently associated with decreased release of both HA and sulphated GAGs [14]. Further pharmacologic studies should be performed to establish the pharmacokinetics and mechanism of 4-MU inhibition of UGDH. Understanding the mechanism of inhibition will help in designing better UGDH antagonists.

Results showing that targeting HA by 4-MU leads to anti-cancer effects *in vivo* show promise that UGDH targeting could have similar anti-cancer treatment. Cancers expressing high levels of UGDH and/or HA could benefit from 4-MU treatment. Given the anti-metastatic potential of targeting UGDH-HA pathway, clinical trials should be established to test the efficacy of 4-MU as a stand-alone therapy or in combination with other chemotherapies or immunotherapies.

## CONCLUSIONS

While much is known about the many roles of UGDH across both normal physiology and oncology, there is still significant work to be done to understand how it can best be harnessed in a clinical setting. Given the potential challenges of directly inhibiting UGDH, therapeutic strategies may extend to targeting downstream pathways and upstream substrates. Additionally, there has recently been more literature published on the potential role of UGDH in treatment resistance and immune modulation across various cancer types. Thus, while directly targeting UGDH may not be feasible as a standalone treatment strategy, it could be an important adjunct to current therapies. Furthermore, modulating its activity could help better understand mechanisms behind drug resistance and/or prevent resistance from developing. Therefore, UGDH is a promising enzyme of interest across many fields of oncology, and given its multi-faceted role in cellular functioning, it plays nuanced and complicated roles within tumorigenic pathways. While these multiple roles can provide challenges, they also provide significant opportunities for therapeutic targeting, prognostication, and drug development.

## Abbreviations

4-MU: 4-Methylumbelliferone
EMT: epithelial mesenchymal transition
GAG: glycosaminoglycans
GBM: glioblastoma
HA: hyaluronic acid
HAS: hyaluronan synthase
PG: proteoglycans
UDP-GlcUA: UDP-glucuronic acid
UDX: UDP-xylose
UGDH: UDP-6 glucose dehydrogenase
